# Pioneering robotic surgery for complex gynecologic conditions: implementation beyond cancer care

**DOI:** 10.1007/s11701-025-02581-1

**Published:** 2025-09-23

**Authors:** Alicia Hernández, Isabel Pascual, Jaime Siegrist, Ana López, Ignacio Zapardiel, Myriam Gracia, Ramón Usandizaga, María del Mar Muñoz, Laura Pérez, Eloy R. Ferreras, Eduardo Alonso, Alicia Carmona, Emanuela Spagnolo

**Affiliations:** 1 Department of Obstetrics and Gynecology, Faculty of Medicine, Universidad Autonoma de Madrid, Madrid, Spain; 2 Endometriosis Unit, La Paz University Hospital, Madrid, Spain; 3 Gynecologic Oncology Unit, La Paz University Hospital, Paseo de la Castellana, 261, 28046 Madrid, Spain; 4 Endometriosis Research Group, Hospital La Paz Institute for Health Research (IdiPAZ), Madrid, Spain; 5 Colorectal Unit, La Paz University Hospital, Madrid, Spain; 6 Pelvic Floor Unit, La Paz University Hospital, Madrid, Spain; 7 La Paz University Hospital, Madrid, Spain; 8 Clinical Development Department, Sinfatin S.L., Toledo, Spain; 9 Department of Obstetric Anesthesia, La Paz University Hospital, Universidad Autónoma de Madrid, Madrid, Spain; 10 Management, Administrative and Economic Area, La Paz University Hospital, Madrid, Spain; 11 Facultad de Medicina, Universidad Autónoma de Madrid, Madrid, Spain

**Keywords:** Robotic surgery, Gynecology, Implementation, Tertiary hospital

## Abstract

Minimally invasive surgery was introduced to minimize some of the issues associated with open surgery. Its benefits include fewer complications, less pain and blood loss, shorter hospital stays, quicker recovery, and less noticeable scars. While laparoscopy has been the gold standard for minimally invasive surgery, robot-assisted surgery has emerged as the latest major breakthrough for minimally invasive procedures, allowing surgeons to expand its use to more complex surgical procedures and operating with more precision, flexibility, control, and comfort than is possible with traditional procedures. Here we present the successful implementation of a robotic-assisted surgery program in the gynecology department of the Spanish tertiary hospital La Paz. This included the complete training of six surgeons from the three units in the department: oncology, pelvic floor, and benign organic pathology. By the end of 2024, 184 procedures had been conducted in the first year, including interventions for both cancer (*N* = 46) and benign pathologies (*N* = 138). In these, 2.2% of patients developed Clavien–Dindo grade 2 complications. The complexity of the procedures increased during implementation of the program, whereas hospitalization times and associated costs remained low.

## Introduction

Minimally invasive surgery allows doctors to perform complex surgical procedures through small incisions, often using specialized instruments and cameras to minimize trauma to the body, with minimal skin and soft tissue incision. Since laparoscopy was introduced in the late 1980s, it has been favored over open surgery in gynecology because it minimizes trauma to tissues, reduces bleeding, reduces the incidence of adhesions and post-operative pain, shortens hospital stays, and speeds up recovery, while improving the appearance of scars [1, 2]. However, it is also associated with a number of limitations and challenges, including a long learning curve for surgeons, counterintuitive hand movements, exacerbation of small movements, or tremors due to the use of long instruments inserted through a fixed entry point, and a limited range of motion, which often requires surgeons to adopt ergonomically challenging positions during procedures. Moreover, 2D optics and the unstable camera platform result in the loss of depth perception and hinder visualization. All these issues can result in fatigue and frustration in the surgeon during lengthy and complex surgeries [3–5].

Robotic surgery has attempted to overcome the limitations of conventional laparoscopic surgery by making surgical interventions more comfortable and precise. Robot-assisted surgery has been performed for over 30 years and its acceptance and implementation are steadily increasing. Robotic systems incorporate improved visualization, with high-definition 3D vision and image magnification, elimination of tremors and involuntary movements and open surgical orientation during instrument movement [6, 7]. The use of a natural position in an ergonomic and comfortable console reduces the surgeon’s physical strain and fatigue, especially during long surgery times, providing a positive impact on the procedure [7]. Today, robot-assisted surgery represents the state-of-the-art in the field of minimally invasive surgery. By combining good training and qualification plan with appropriate patient selection, robotic surgery can be highly advantageous, reducing blood loss, lowering conversion rates and shortening hospital stays [4, 8, 9]. Despite this fact, data on its utility and outcomes are often limited or controversial when making comparisons with traditional laparoscopic techniques [5, 10–12]. However, post-operative quality of life (measured on a linear scale from 0 to 100) has been reported as significantly higher when comparing robotic procedures with laparoscopy [13]. Nonetheless, there is still a clear demand for high-quality randomized trials to objectively quantify the benefits of its use [10, 14, 15].

While its application in gynecologic surgery has been slower than in other specialties, like urology, the number of indications is continuously and rapidly growing [7]. Robot-assisted surgery is currently used in different benign [16] and malignant gynecologic conditions [17], such as myomectomy, endometriosis surgery, sacrocolpopexy, lymphadenectomy, and sentinel lymph node dissection, with similar or better outcomes than with laparoscopy [2]. According to data provided by ABEX, the company that markets the da Vinci system in the Iberian Peninsula, the aforementioned system is mainly used for oncology procedures (76% of the total), followed by endometriosis resection (15%), ovarian and fallopian tube surgery (4%), benign hysterectomies (2%), myomectomies (1%), and others.

The use of robotic surgery allows minimally invasive surgery to be extended to a wider range of gynecological patients (such as obese patients) and to challenging procedures that hitherto have not been addressed with classic laparoscopic techniques. In endometriosis, the adhesive nature of the disease, with obliteration of the surgical planes, complicates dissection of all endometriotic implants. Moreover, straight-stick laparoscopy limits surgeons in reaching the corners of the pelvis, while the Da Vinci system has increased handling and arm, and the amplitude of instrument movement with 7 degrees of freedom. In this environment, all new developments and technology provided by robot-assisted surgery can be considered especially useful for increasing the precision of dissection in endometriosis and improving the identification and preservation of the autonomic nerves, providing better functional results.

Despite the consensus over the net benefits of robotic assistants, there are still challenges that hinder their implementation. The most relevant is still probably the expense associated with the equipment, especially the initial investment in the robot (around €2 M) and its annual maintenance (around € 120,000) with an estimated cost of about € 2000/procedure for disposable equipment [14].

La Paz University Hospital is a third-level public hospital complex, dependent on the Community of Madrid, located in the northern part of the city. It provides coverage for over 500,000 inhabitants of Madrid and other Spanish regions. In 2023, it employed 1266 physicians and 1855 nurses and had 1187 beds and 48 operating rooms. Below we describe the implementation process, experiences and outcomes from the first 15 months of use of a robotic surgery system in the gynecological department of this Spanish tertiary hospital.

## Materials and methods

### Process planning

#### Definition of objectives and scope of the robotic surgery program

After planning the acquisition of the robotic surgery system, a structured implementation program was designed to integrate its benefits into gynecologic surgery. The main objective was to enhance surgical precision, reduce complications, and improve patient outcomes, with special focus on patients who would benefit the most, such as those with deep infiltrating endometriosis, gynecologic malignancies, and comorbidities such as obesity. To ensure a safe transition, a multidisciplinary team developed clinical protocols, patient selection criteria, and a structured surgeon training program.

#### Implementation timeline

The program was implemented in sequential (although often overlapping) phases, allowing for progressive adaptation of the hospital infrastructure, surgical teams, and patient care protocols. While some phases had specific targets, others overlapped to ensure a continuous and efficient integration process.

Phase 1: Planning and Infrastructure Set-up (January–July 2023).

- Acquisition and installation of the robotic system.
- Development of clinical protocols and patient selection criteria.
- Institutional approvals (hospital administration and ethics committee).

Phase 2: Surgeon Training and Team Preparation (March–September 2023).

- Theoretical and practical training of first surgeons and OR staff.
- Simulation sessions and hands-on practice with the robotic system.
- Supervised initial cases under external proctoring (July–September 2023).

Phase 3: Initial Clinical Implementation (October 2023–June 2024).

- First robotic operations performed under supervision.
- Gradual increase in complexity while refining surgical protocols.
- Continuous monitoring of perioperative outcomes.

Phase 4: Full Integration and Evaluation (Months 10–18, overlapping with clinical implementation).

- Independent robotic operations by certified surgeons.
- Comprehensive evaluation of outcomes, efficiency, and patient benefits.
- Final assessment and optimization of the program for long-term sustainability.

#### Selection of the robotic surgical system and necessary infrastructure

The selected robotic system, the da Vinci Xi, comprises three primary components: the surgeon’s console, which provides hand and foot controls to manipulate the robot’s instruments; the patient cart, with four robotic arms to hold surgical instruments; and the 3D HD Vision System, which provides a high-definition, three-dimensional view of the surgical field.

The system is equipped with a dual console, so two surgeons can operate simultaneously, thus allowing for a primary surgeon and a trainee or two surgeons from different clinical specialties to work together during a single procedure.

#### Institutional approvals and ethical considerations

The program was developed as part of the regional strategic plan of the Community of Madrid for implementing robotic surgery across six major hospitals. It was aligned with hospital management guidelines to maintain high-quality standards. Ten timeslots were allocated each month to integrate robot-assisted surgery into routine department activities.

#### Multidisciplinary team training

A specialized robotic surgery training program was designed for all the personnel involved, including gynecologists, anesthesiologists, and nurses.

None of the gynecologists in the department had prior experience with robotic surgery. Therefore, only highly experienced surgeons, with more than 10 years of advanced laparoscopic surgery experience, were selected to participate in the initial phase of the program.

The training program adhered to ABEX guidelines and recommendations for a standardized educational curriculum in robot-assisted gynecological surgery as provided by the Society of European Robotic Gynaecological Surgery [13]. The program included online training, simulation cases, case observations, training with cadavers, procedural video training, assisted procedures, and performing non-assisted, recorded cases.

The training process consisted of four main steps:

1. Introduction to the da Vinci technology, including online training and videos, conducted prior to system setup.
2. Post-setup workshops focusing on suturing, energy, dissection, and use of the da Vinci simulator, along with certification at the Intuitive Centre.
3. Initial cases with expert tutor support.
4. Ongoing updates, including support for new indications and advanced courses.

The dual console setup allowed surgeons to simultaneously observe operations during their training, thus fostering collaborative learning. The learning curve was established at around 20 interventions for the console surgeon and 10 for the bed assistant surgeon. Once established, the two surgeons remained unchanged throughout the whole implementation program.

A dedicated nursing team was selected to provide assistance during robotic procedures. All the nurses took a specific training program before the start of the implementation phase. The nurses remained unchanged throughout the process.

### Set-up

In the operation room, the consoles from which the surgeons operated the robot were always placed on the right side of the patient.

The system configuration was:

- Arm 1: Maryland bipolar forceps
- Arm 2: optics (0º or 30º)
- Arm 3: monopolar scissors
- Arm 4: Cadiere grasper

Sealers were used for omentectomies only.

Trocar placement: the robotic ports were aligned along the patient’s abdomen. The optical trocar was inserted through the umbilicus. A trocar serving as an accessory port was positioned on the left side, opposite arms 1 and 2, in a triangular configuration.

### Patient selection

#### Inclusion and exclusion criteria for robot-assisted gynecologic surgery

Patients were selected from those scheduled for gynecologic laparoscopic surgeries within the hospital Gynecology Department.

At the start of the program, preference was given to low-complexity procedures, such as those involving patients with no prior surgery or with ASA scores of up to 3, as well as patients with low-risk or no intestinal resection requirements in endometriosis, and those with early-stage endometrial cancer. As the surgeons gained experience and confidence, more complex procedures were gradually incorporated, including deep endometriosis with bowel involvement and radical hysterectomy for early-stage cervical cancer.

The complexity of procedures was categorized as follows:

- **Low-complexity procedures**:
  o Adnexectomy for non-endometriotic adnexal mass
  o Cystectomy for non-endometriotic adnexal mass
- **Intermediate complexity procedures**:
  o Procedures not classified as low or high complexity, including hysterectomies not associated with deep infiltrating endometriosis.
- **High-complexity procedures**:
  o Myomectomy
  o Oncologic surgery with omentectomy or lymph node dissection
  o Sacrocolpopexy
  o Deep endometriosis with bowel or urinary involvement with or without hysterectomy

#### Preoperative evaluation and risk assessment

Preoperative evaluations and follow-up care were performed in accordance with hospital standards and relevant guidelines.

#### Informing and obtaining informed consent from patients

All selected patients were fully informed about the surgical procedure, the associated risks and benefits of robotic surgery, and gave their written consent voluntarily prior to the operation.

### Post-operative follow-up

All patients were monitored after the surgery as per hospital standards for equivalent laparoscopic procedures, including short- (before discharge) and long-term follow-up. All relevant data were recorded in the patient’s medical records. Post-operative complications were graded according to the Clavien–Dindo classification. Recovery time before discharge, readmission and surgical success rates were collected.

### Data collection and analysis

Data were collected in the patients’ clinical records following normal clinical practice and the Hospital’s procedures. All relevant data were later pseudo-anonymized and recorded in a Microsoft Excel spreadsheet for descriptive statistical analysis. Data analysis was performed by surgeons and assistants in the gynecology department. For quantitative variables, mean, standard deviation, and minimum and maximum values were calculated. For qualitative variables, number and percentages were calculated.

## Results

After setting up the da Vinci system, 184 robot-assisted procedures were performed in the period from July 2023 to December 2024. All patients were adult women with a mean age of 45.5. There was wide age variability, ranging from 18 to 80 years.

**Fig. 1:**
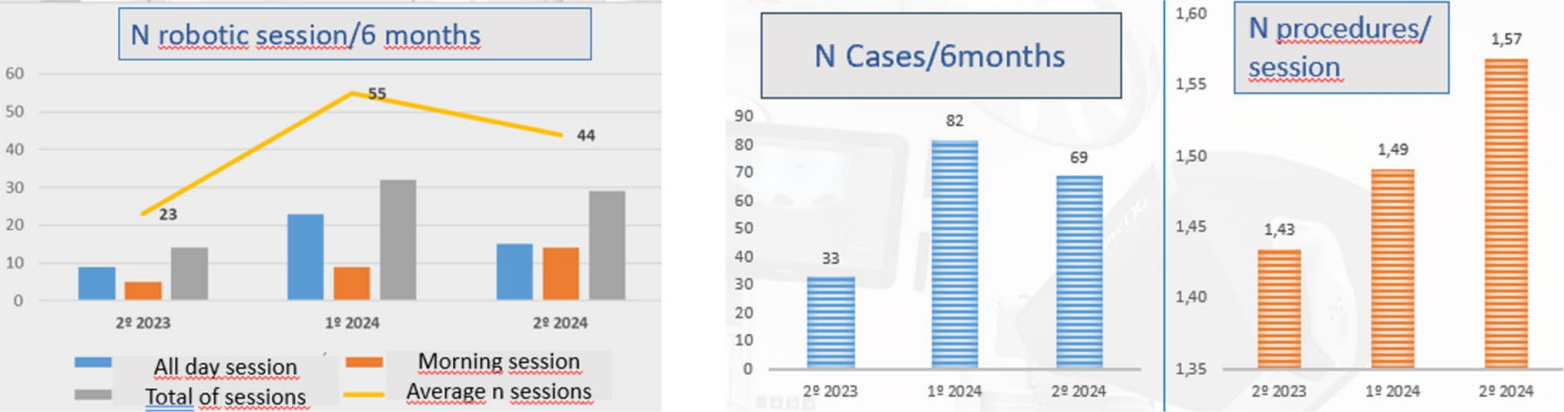
Number of sessions and cases

There were on average 10 robot-assisted surgery sessions a month, 2 every Thursday and alternate Fridays. There were 23 sessions in 2023 and 55 and 44 sessions in the first semester and second semester of 2024, respectively. While the number of sessions and cases decreased slightly in the second half of 2024 (82 in the first half vs 69 in the second half), the number of procedures per session steadily increased from the beginning of the program, from 1.43 in 2024 to 1.57 in the last semester of 2024, showing increasingly better use of the resources as the program advanced **(**Fig. 1**)**.

### Certification programs and the nursing team

During the implementation, six console surgeons were trained, two from the gynecology oncology unit, two from the endometriosis unit, and one from the pelvic floor unit. Additionally, three surgery assistants took the training program and were allowed to help the surgeons. By the end of 2024, four had completed their learning curves as first surgeon. The nurses were trained and remained unchanged throughout the program.

### Indications

Among the 184 robot-assisted gynecologic operations, 138 (78%) were performed for benign pathologies and 46 (26%) for oncologic pathologies. A summary of the surgical indications is shown in Table 1.

**Table 1:** Surgical indications 2023–2024

Cancer	Endometrial cancer	28	(15%)
	Cervical cancer (in situ)	16	(9%)
	Borderline ovarian tumor	2	1%
Benign pathology	Endometriosis	46	(25%)
	Myomas	48	(26%)
	Adnexal mass	32	(18%)
	Sacrocolpopexy	2	(1%)
	Other (Lynch sd, AUB)	10	(5%)

**Fig. 2:**
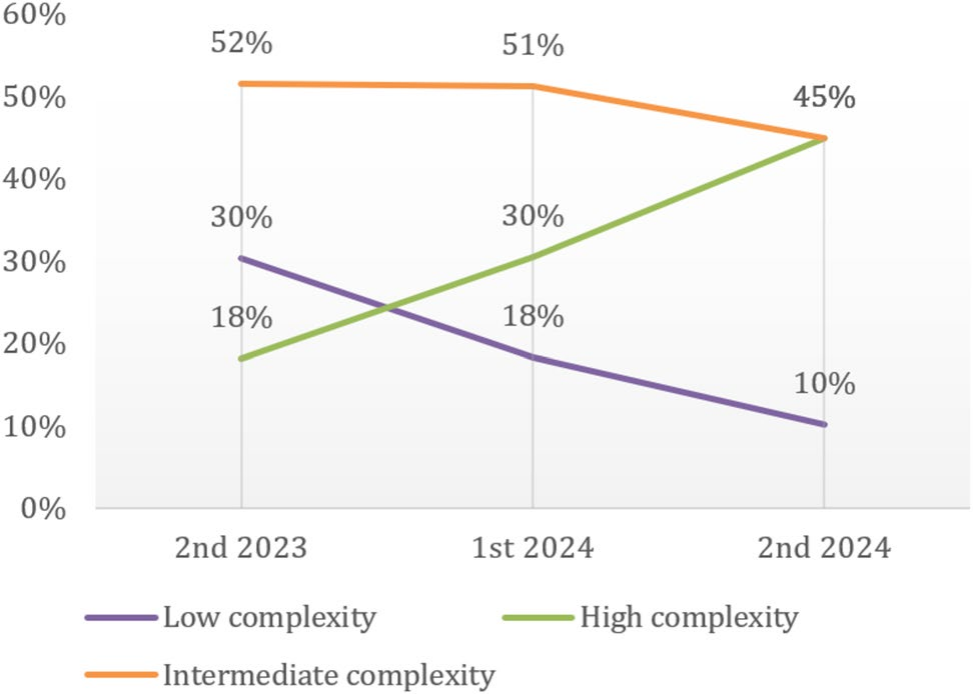
Evolution of the complexity of interventions

The strategy for the implementation of the program involved starting with the less complex procedures, prone to few post-operational complications, then increasing the complexity as the program progressed, and the surgeons and the teams gained experience and confidence in the use of the system (Fig. 2).

Over the first year of implementation, the low-complexity/high-complexity procedure ratio shifted to more complex procedures, covering those patients who would benefit the most from the advantages of robotic assistance as the surgeons gained experience. In 2023, 33 procedures were conducted, 10 (30%) of which were considered low-complexity procedures and 6 (18%) as high complex procedures. During the later stages of the program, in the second semester of 2024, out of the 69 procedures conducted, 7 (10%) were considered low-complexity and 31 (45%) high-complexity procedures. Almost half of the procedures (48.9%) fell outside this classification, being either considered low or high complexity.

### Procedures

The surgical procedures performed during the implementation process are described in Table 2.

**Table 2:** Interventions performed during the first 15 months implementation process

Procedure	*n*	%
*Gynecologic cancer*		
Hysterectomy ± adnexectomy	16	35%
Hysterectomy + Sentinel lymph node	28	60%
Staging Borderline OT + omentectomy	2	5%
*Endometriosis*		
Endometrioma	19	41%
Deep Infiltrating Endometriosis	23	50%
Endometrioma + Deep Infiltrating Endometriosis	4	9%
*Myomas*		
Hysterectomy	38	79%
Myomectomy	7	15%
Multiple myomectomy	3	6%

**Fig. 3:**
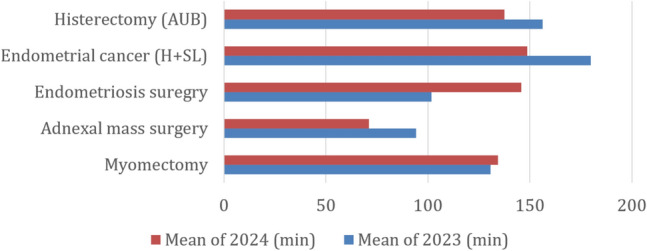
Mean intervention times per procedure in 2023 and 2024

The mean duration of the procedures (excluding the two procedures that required conversion) was 125 min (console time), ranging from 30 to 300 min, with a mean time of 73.3 min for low-complexity surgeries and 154.9 min for the most complex procedures. The docking time for each session was about 12–20 min. Mean times were 123.8 min (ranging from 49 to 230) in 2023, 124.6 min in the first semester of 2024 (from 40 to 292 min), and 126.9 min (ranging from 30 to 300 min) in the second semester of 2024. Considering the data over time, the mean procedure time decreased for almost all procedures when comparing the mean times for 2023 and 2024 (Fig. 3). The only two exceptions were myomectomies, where the length of the procedures remained almost unaltered, and endometriosis surgery, in which there was a significant increase.

### Cost-efficacy

**Fig. 4:**
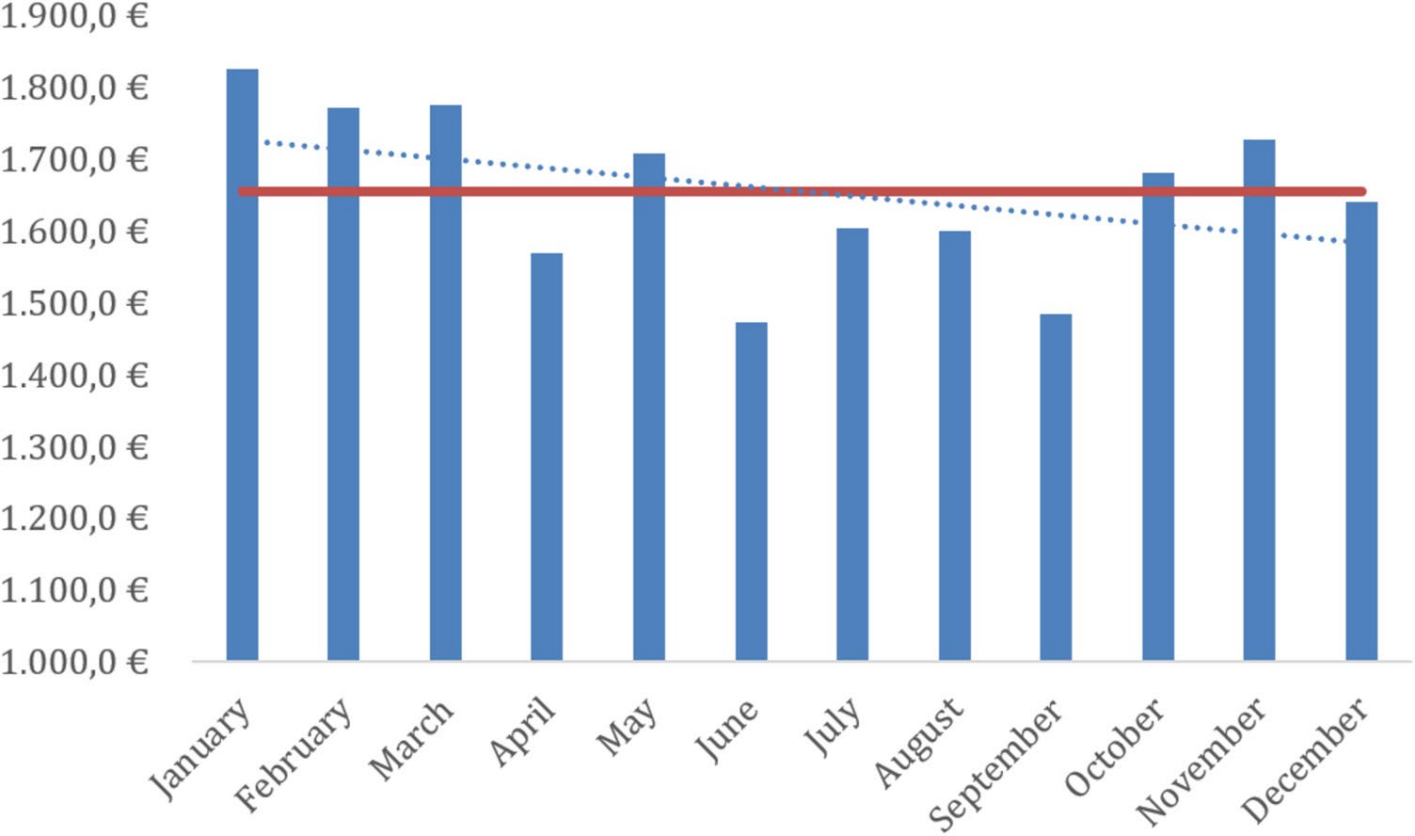
Mean intervention costs for 2024

Figure 4 shows the mean costs of using the system in 2024. In 2024, this was €1654.33 (slightly lower than in 2023, when the cost was €1948.00), ranging from €1472.00 to €1824.00. Although there were no significant differences, there was a slight downward trend in the cost even as the rate of more complex procedures increased, due to better tool use. The costs considered here include only those derived from the use of the robot.

Regarding indirect costs (Operating Room overhead, medical staff, facilities costs), the mean cost was € 3561.00. The Average Cost per Procedure was € 5215.00.

Mean Cost per Hospital Stay (1 day) was € 1257 and no cases of readmission due to complications have occurred.

### Outcomes

There were no intra-operative complications during the 184 surgeries performed. Four cases (2.2% of the total procedures) presented non-serious post-operative complications, such as surgical wound infection requiring antibiotic treatment (Clavien–Dindo = 2).

Two cases required conversion due to the size of the uterus, which extended above the navel, giving an overall conversion rate of 1.1%.

**Fig. 5:**
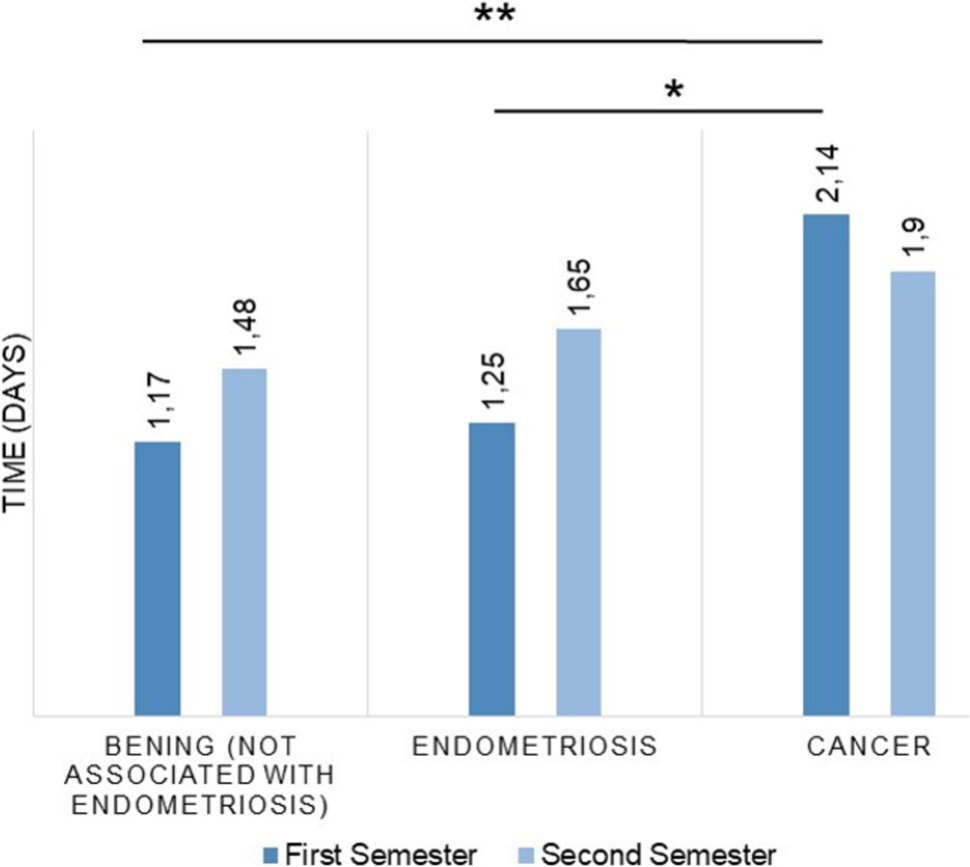
Mean hospitalization time per semester (2024)

The mean hospitalization time was 1.5 days (from 0 to 8 days) with the average for low and high complex surgeries being 0.3 and 2 days, respectively. Average hospitalization time was significantly higher for cancer surgery than benign pathologies or endometriosis operations. In 2024, the hospitalization period increased for all procedures except cancer surgery, which dropped from 2.14 to 1.9 days after surgery. Figure 5 shows the mean hospitalization time during the first and second semesters of the year 2024.

## Discussion

We present here one of the few reports detailing the process of implementation of robotic surgery in the gynecology department of a tertiary Spanish hospital and the results of the first year of activity.

To achieve the best results when implementing robotic surgery, it is important to establish policies and criteria that include careful surgeon selection, the training needed to obtain essential skills, case volume, procedures, patient outcomes, and the specialties that will be given access to the technology. A very specific training program for personnel is needed to ensure patient safety, optimal outcomes, and the correct use and leveraging of the technology. As described in previous works, one of the advantages of robotic systems is the shorter learning curve and a less stressful process compared to learning other types of minimally invasive surgery, such as laparoscopy [11]. Furthermore, surgeons perform better in drills compared to laparoscopy [18]. At the beginning of the program, there were no accredited surgeons in the department. By the end of 2024, six surgeons and three assistants were certified to perform surgery using the da Vinci system.

Our approach involved three units in the gynecology department: oncology (the best-established gynecologic field), benign organic pathology, and pelvic floor. In public hospitals with similar characteristics, robot assistants are predominantly used for minimally invasive oncology procedures, especially during implementation programs [2]. In our experience, the program was designed to consider all interventions and patients that would potentially benefit the most from the use of the robotic systems.

Regarding the cases themselves, the implementation plan involved starting with procedures that were less complex and less prone to post-operational complications. As the program progressed and the surgeons and teams gained experience and confidence in the use of the system, the complexity of the procedures gradually increased, always with patient safety as the main criterion for decision-making during the process. According to ABEX, endometriosis procedures, which are often highly complex, account for only 15% of the main operations performed with the da Vinci robot, whereas they represented up to 25% of our procedures (*N* = 46) during the implementation process. Myomectomy is also considered one the procedures that can most benefit from the use of robot-assisted surgery, due to its complexity, which requires careful and extensive suture. Robotic technology provides the same advantages of minimal invasiveness as laparoscopic surgery, while also offering improved stability and maneuverability than with open surgery. In the case of myomectomies, which generally only account for around 1% of procedures (ABEX data), we conducted 10 operations, representing 5.4% of the total.

Our approach allowed us to fully exploit the investment and maximize the number of patients benefiting from the program, without causing any disturbances or delays in the normal functioning of the department. It ensured smooth implementation of the program and guaranteed the safety of patients while the surgeons gained experience and confidence with the system.

Overall, neither safety issues, increase in the number of complications, nor their severity were observed at any time during the first year using the robotic assistant. There were no serious complications during or after the procedures and the overall conversion rate during the program was low (1.1%), in line with previous studies [9]. Conversion rates have been shown to drop while gaining experience [9, 19], and this will likely occur at our hospital. With a stable team of qualified surgeons and assistants, the integration of new staff in the department is expected to be shorter and easier while maintaining quality standards.

The double console, where two specialists can work together in the same session, was not only relevant during training sessions, where a trainee can follow the procedures while a more experienced and accredited surgeon performs them, but was also essential for more complex procedures as it allowed two surgeons from two different specialties to perform the surgery during the same session as was the case with endometriosis involving the intestine [20, 21].

In relation to operating times, previous studies are inconclusive regarding the benefits of robotic assistants over laparoscopic surgery, with some reporting longer times when using the robotic system and others reaching the opposite conclusion, while some found no significant differences [2]. In our case, times ranged from 30 to 320 min, with a mean time of 73.3 min for low-complexity and 159.9 min for high-complexity surgery. A reduction in operating times was observed for almost all procedures as the program developed. Two exceptions were myomectomies, which remained unaltered, and endometriosis surgery, which showed a significant increase in operating times. It is important to note that, as stated above, the complexity of the procedures increased as the program progressed, particularly in the case of endometriosis surgery, which could explain longer procedure times. The rest of the procedures, even when including more complex cases, had shorter surgery times, mainly due to the experience gained by the surgeons and their assistants as they performed more procedures.

The mean hospitalization time was 1.5 days, ranging from 0 to 4 days. The two conversions are not included in calculating these times. As expected, the times were significantly higher for cancer surgery than for benign pathologies or endometriosis. Over the first year, the hospitalization time increased for all procedures except cancer surgery, which dropped from 2.14 to 1.9 days. This rise can also be explained by the greater complexity of the procedures.

The significant costs, especially the large initial investment needed to purchase a robotic system, can be discouraging and deter the decision to opt for these systems. However, this can be partly offset by extending the use of the robot. In our case, the large population that Hospital La Paz covers allowed us to reach a high number of patients, especially endometriosis patients. We performed almost 200 operations in the first year, improving the cost–benefit ratio, and we expect to increase the number in the near future. Besides the initial investment, some studies point out that longer operating times could explain the high cost of the procedures [22]. In our case, we showed that well-trained and experienced surgeons can achieve very reasonable operating times after a short learning curve, even for the most complex procedures.

Furthermore, the robot makes minimally invasive surgery an option available to more patients, such as obese patients or patients with a large uterus and previous surgery/adhesions, and for deep endometriosis [20, 21].

A thorough analysis of the cost-effectiveness of a system should take into consideration not only the direct costs of the surgery, but also indirect costs dependent on other variables, such as length of hospitalization, blood loss and other complications, readmissions, mortality, and surgery time. Our results show consistent costs for the procedures in the first year with a slight downward trend due to more efficient use of the machine and its components. However, our work does not take into consideration other indirect costs arising from these kinds of procedures as a thorough cost–benefit analysis was beyond the scope of this project. Some studies have tried to establish whether, in economic terms, the costs of these systems are compensated in other areas, and found that the number of interventions performed and the inclusion of all aspects related to life-long patient health are relevant variables in obtaining a favorable return [23, 24].

There is still a need of stronger scientific evidence, with randomized clinical trials to develop evidence-based practices for cost restraint in robotic surgery and to unequivocally measure the advantages of robot-assisted surgery.

### Limitations

There are two main limitations to this study: the first is a lack of a comprehensive comparative analysis with analogous laparoscopic procedures to determine the relative benefits of the use of the Da Vinci robot. As the main objective of the study was to introduce the plan, procedures, and methods for the implementation of the robot assistant in a tertiary public hospital in Spain, the main analysis is descriptive and lacks comparisons. The second main limitation is the absence of indirect costs in the economics analysis; only the direct costs of using the system were included in the data analysis and discussion. Further analysis is needed to assess the relative safety, efficacy, and cost-effectiveness of the robotic system in comparison with other procedures used for the treatment of these patients in the three units involved in the program.

### Challenges

Despite the general consensus over the advantages of the robotic surgery systems versus other procedures, implementation is slow and its use is mainly restricted to the most complex gynecologic procedures [25]. The complexity of the robotic system can also lead to technical challenges during surgery, such as malfunction or technical issues. Some other challenges that have to be addressed are: integration of both robotic and laparoscopic surgeries in the training programs; communication between the surgeon and the team; the need for a dedicated space to install the machine; the need for a stable, qualified team; and even reluctance among some surgeons to adopt and adapt to new procedures and among managers due to the high initial investment [26]. Adapting the whole team is essential for the implementation of the robotic surgery.

## Conclusions

A well-established training program, a stable team (including surgeons and nurses), and a carefully structured plan were crucial to successfully implementing and running a robot-assisted surgery system in the gynecology department of a Spanish tertiary hospital. Quality standards were assured with no safety issues, and little or no interference with the normal functioning of the service.

The considerable initial expenses seem to be compensated by the extensive use of the robot and the broad range of conditions and variety of procedures that can be approached.

The data from the first year suggest a positive balance in the implementation of these systems, which are likely to improve even further in the near future in terms of a number of patients and a variety of procedures.

## Data Availability

No datasets were generated or analyzed during the current study.

## Abbreviations

AUB: Abnormal Uterine Bleeding
H: Hysterectomy
IdiPAZ: Hospital la Paz Institute for Health Research
SL: Sentinel Lymph
